# An Integrated Strategy to Study Muscle Development and Myofilament Structure in *Caenorhabditis elegans*

**DOI:** 10.1371/journal.pgen.1000537

**Published:** 2009-06-26

**Authors:** Barbara Meissner, Adam Warner, Kim Wong, Nicholas Dube, Adam Lorch, Sheldon J. McKay, Jaswinder Khattra, Teresa Rogalski, Aruna Somasiri, Iasha Chaudhry, Rebecca M. Fox, David M. Miller, David L. Baillie, Robert A. Holt, Steven J. M. Jones, Marco A. Marra, Donald G. Moerman

**Affiliations:** 1 Department of Zoology, University of British Columbia, Vancouver, British Columbia, Canada; 2 Michael Smith Genome Sciences Centre, British Columbia Cancer Agency, Vancouver, British Columbia, Canada; 3 Department of Cell and Developmental Biology, Vanderbilt University Medical Center, Nashville, Tennessee, United States of America; 4 Department of Molecular Biology and Biochemistry, Simon Fraser University, Burnaby, British Columbia, Canada; Stanford University Medical Center, United States of America

## Abstract

A crucial step in the development of muscle cells in all metazoan animals is the assembly and anchorage of the sarcomere, the essential repeat unit responsible for muscle contraction. In *Caenorhabditis elegans*, many of the critical proteins involved in this process have been uncovered through mutational screens focusing on uncoordinated movement and embryonic arrest phenotypes. We propose that additional sarcomeric proteins exist for which there is a less severe, or entirely different, mutant phenotype produced in their absence. We have used Serial Analysis of Gene Expression (SAGE) to generate a comprehensive profile of late embryonic muscle gene expression. We generated two replicate long SAGE libraries for sorted embryonic muscle cells, identifying 7,974 protein-coding genes. A refined list of 3,577 genes expressed in muscle cells was compiled from the overlap between our SAGE data and available microarray data. Using the genes in our refined list, we have performed two separate RNA interference (RNAi) screens to identify novel genes that play a role in sarcomere assembly and/or maintenance in either embryonic or adult muscle. To identify muscle defects in embryos, we screened specifically for the Pat embryonic arrest phenotype. To visualize muscle defects in adult animals, we fed dsRNA to worms producing a GFP-tagged myosin protein, thus allowing us to analyze their myofilament organization under gene knockdown conditions using fluorescence microscopy. By eliminating or severely reducing the expression of 3,300 genes using RNAi, we identified 122 genes necessary for proper myofilament organization, 108 of which are genes without a previously characterized role in muscle. Many of the genes affecting sarcomere integrity have human homologs for which little or nothing is known.

## Author Summary

Muscular diseases affect many people worldwide. While we have learned much about the sarcomere, the basic building block of muscle cells, there are still numerous questions that remain to be answered. We must learn more about proteins expressed in muscle and how they interact so that better treatments for myopathies can be developed. The nematode *Caenorhabditis elegans* is a valuable model organism for the study of muscle due to similarities between worm body wall muscle and vertebrate muscle, along with its semi-transparent cuticle that allows for visualization of muscle structures in live animals. We have used transcriptional profiling methods to identify the majority of genes that are expressed in the embryonic body wall muscle cells of *C. elegans*. To gain insight into possible functions performed by these genes and their corresponding proteins, we examined animals and muscle cells for abnormalities after the targeted inactivation of about 3,300 genes. We identified 122 genes necessary for proper myofilament organization, 108 of which had no previously characterized role in muscle. This approach proved to be a rapid and sensitive means to identify genes that affect muscle differentiation and sarcomere assembly.

## Introduction

Muscle tissue is important for humans in a myriad of processes including movement, digestion, and the pumping of blood through the cardiovascular system. Afflictions that affect muscle can be debilitating in any of these processes. The underlying cause of many myopathies lies within the cells that make up muscle tissue. Specifically, defects in components of the functional repeat unit of muscle, the sarcomere, are implicated in over 20 diseases [1]. The nematode *Caenorhabditis elegans* is a valuable model organism for the study of muscle due to the similarity of worm body wall muscle to vertebrate muscle, along with its semi-transparent cuticle that allows for visualization of muscle structures *in vivo*. The basic protein components within a *C. elegans* sarcomere have vertebrate counterparts, with only minor differences in protein composition and organization [2]–[4]. Much of the work focused on sarcomere assembly is restricted to muscle cells that contain multiple sarcomeres and make up body wall muscle in *C. elegans*. Building a functional sarcomere in *C. elegans* is a complex process that requires the assembly of two main attachment complexes, the M-line and dense body. Both of these structures are anchored in the sarcolemma, projecting inwards to allow for anchoring of actin filaments in the case of dense bodies, or myosin filaments in the case of the M-line. This anchorage is necessary to transmit the force created from the contraction of myofibrils to the muscle cell basement membrane [2],[5]. Many of the proteins needed to form a functional sarcomere in the worm are homologs of proteins required in vertebrate focal adhesion complexes [3]. Adhesion complexes are found in migrating cells involved in a number of processes including tissue repair, immune responses, and tumor formation [6].

Much of what we know about the protein composition of adhesion complexes in *C. elegans* muscle was uncovered via mutational screens. Uncoordinated movement (Unc) and Paralyzed and Arrested at Two-fold stage (embryonic arrest, Pat) are the two primary phenotypes used to identify muscle mutants in the worm. An Unc phenotype can be caused by a defect in muscle cells, the neural architecture or neuronal function which triggers their contraction. The Pat phenotype is more severe and appears to be primarily muscle specific in nature, as almost all Pat mutants identified by Williams and Waterston (1994) [7] have since been shown to be components of the attachment complex or associated with the basement membrane. All of the proteins linked to this phenotype are essential for the initial assembly of the sarcomere and/or involved in the attachment of muscle cells to the basement membrane [7],[8], including UNC-52/perlecan [9], PAT-3/β-integrin [10], PAT-4/ILK [11] and DEB-1/vinculin [12]. Proper muscle function, attachment to the basement membrane and communication with the underlying hypodermis are critical for elongation of embryos beyond the two-fold stage [7]. Interestingly, less severe mutations in some Pat genes lead to an Unc phenotype due to partial function of the protein. The *unc*-*89*, *unc*-*95* and *unc*-*98* genes also encode proteins found in the attachment structures that anchor the myofilament lattice [13]–[15]; however, loss of their gene products is associated with an Unc, rather than a Pat, phenotype. Animals homozygous for null mutations of another gene class, the Dim (disorganized muscle) mutants, exhibit a far less acute phenotype. These animals are wild type in appearance and movement, but display minor muscle defects upon close observation [16].

The work done in *C. elegans* first demonstrated the important role of the basement membrane protein UNC-52/perlecan in the initial assembly of adhesion structure in the muscle cell membrane [8],[9],[17] as well as the key role of the UNC-112 protein and, by inference, its human orthologs Kindlin-1,2,3 in the assembly of integrin-containing attachment complexes [18],[19]. Furthermore, studies on flies and worms demonstrate that ILK is an adaptor molecule and all of its behavior in regards to muscle attachment can be explained as an adaptor molecule, not as a kinase [11],[20],[21]. The many studies of adhesion receptors that have been done over the years have led to the idea that adhesion complexes are platforms where many molecules meet to organize the cytoskeleton and trigger signalling. Recent studies highlight the striking similarity between vertebrate focal adhesion plaques and *C. elegans* muscle adhesion structures and position integrin linked kinase, as well as FERM and LIM domain proteins as central players at focal adhesions [3],[22].

In humans, mutations in several sarcomeric and sarcolemmal proteins have been shown to cause muscular dystrophy and cardiomyopathies (reviewed in [23]). For example, loss of Kindlin-1, an actin-ECM linker protein first described in *C. elegans* as UNC-112 [18], causes Kindler syndrome, characterized by neonatal blistering, sun sensitivity, muscle atrophy, abnormal pigmentation, and fragility of the skin [24]. Clearly, an understanding of muscle development and function is an important step towards the development of treatments for muscle-related disease.

To date, mutational screens for genes affecting *C. elegans* body wall muscle have identified about 70 genes. Recent studies using DNA microarray technology have identified thousands of transcripts that are made in muscle cells. Using this method of transcriptional profiling Roy et al. [25] were able to identify 1,364 genes enriched in the body wall muscle of L1 larvae, and Fox et al. [26] were able to identify 5,710 genes expressed in embryonic body wall muscle. Another method of identifying large numbers of transcripts from isolated tissue specific RNA is Serial Analysis of Gene Expression or SAGE [27]. This technology differs from gene chip technology because it allows the identification of all transcripts including those not previously identified by analysis of the sequenced genome. Here, we use this technology to identify over 7,900 genes that are potentially expressed in embryonic muscle cells. Several large-scale transcriptional profiling screens using DNA microarray technology and more recently RNA-Seq have been done on various mouse and human tissues and cell lines including muscle [28]–[30]. In this study we describe the first SAGE gene expression profile done on muscle tissue in *C. elegans*.

In addition to generating a list of genes expressed in embryonic muscle cells, we have carried out two RNAi screens designed to identify muscle-affecting genes in *C. elegans*. Screening was performed using a subset of about 3,600 genes that were found to be expressed in muscle using both SAGE and microarray analysis [26] and thus have a high probability of being expressed in body wall muscle. In order to find as many muscle affecting genes as possible, our screens were designed to identify myofilament-affected animals, regardless of whether the worms exhibited a visible phenotype or appeared wild type. We have identified 122 genes involved in proper myofilament organization, 108 of which had no previously characterized role in muscle structure or function.

## Results

### An embryonic-muscle transcriptome for *Caenorhabditis elegans*

**Figure 1:**
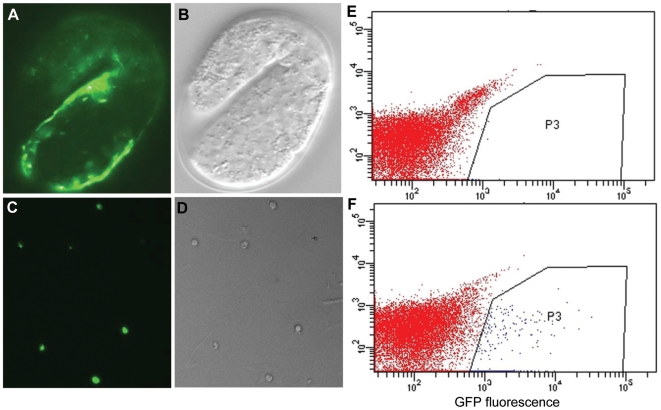
GFP-tagged embryonic muscle cells purified by FACS. MYO-3::GFP expression in the body wall muscle cells of an RW1596 embryo (A,B), and in purified embryonic muscle cells after isolation and FAC sorting (C,D) visualized by Fluorescence microscopy (A,C) and DIC microscopy (B,D). Final fluorescence intensity scatter plot of wild-type (non-GFP) cells (E) and MYO-3::GFP positive cells (F). Cells in box P3 were sorted as GFP positive.

The *C. elegans* embryo has a total of 123 muscle cells, the majority of which (81) are body wall muscle cells [31]. The *myo*-*3* gene encodes a myosin heavy chain protein which is the central component of body wall muscle thick filaments and is present in at least 85 embryonic muscle cells [32]. Myosin protein expression is first detected during mid embryogenesis. Tagging MYO-3 with GFP and expressing it under the control of its own promoter is therefore an excellent way of identifying muscle cells prior to and during early myofilament formation using GFP fluorescence [33] (see Figure 1A and 1B).

We have used a strain expressing a GFP-tagged MYO-3 protein [34] to obtain a relatively purified population of embryonic muscle cells after Fluorescence Activated Cell Sorting (FACS). Whole embryos were manually disrupted to obtain a suspension of mostly single cells that was then passed through a FACS machine to collect viable GFP expressing cells (see Figure 1C–1F). Typically, we obtained a range of 90% to 95% GFP expressing cells at the final step. Only samples that consisted of at least 90% fluorescing MYO-3::GFP live cells were used for RNA extraction.

**Figure 2:**
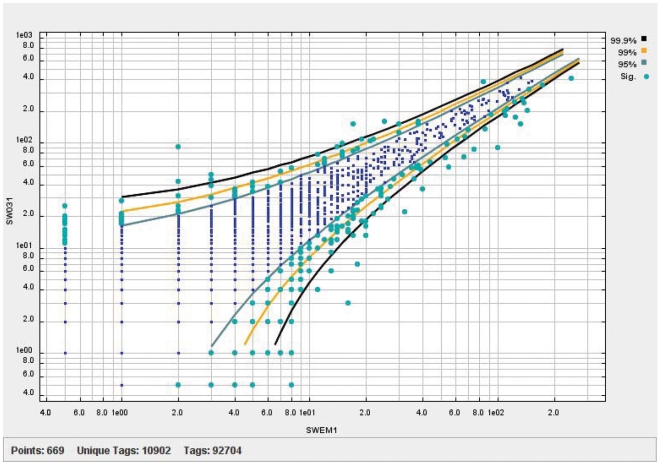
Reproducibility of SAGE biological replicate libraries. Comparison of two Long SAGE biological replicate libraries for embryonic muscle cells in *C. elegans* (SW031 and SWEM1). A total of 88% of the sequence tags are similarly expressed with a correlation coefficient of 0.94. DiscoverySpace was used to generate the plot [68].

We have constructed two Long SAGE biological replicate libraries using RNA isolated from our FAC-sorted embryonic muscle cells (data available at http://elegans.bcgsc.bc.ca). A total of 49,655 and 120,825 sequenced tags were obtained from the SWEM1 and SW031 libraries, respectively. We used a sequence quality filter of 0.99 to reduce the number of tags incorrectly assigned due to sequencing errors. This gave us adjusted tag numbers for the two libraries of 33,827 and 89,561, respectively and these numbers were used for all subsequent analyses. By combining the data from both libraries we identified 10,724 unique sequence tags representing 7,974 genes expressed in embryonic muscle cells. Despite the difference in sampling size between these two libraries almost 88% of the genes identified are present in both SWEM1 and SW031. In addition, the correlation coefficient of tag counts observed for genes found in the two libraries is 0.94, indicating that variability between these samples is low (Figure 2). This result is comparable to the correlation coefficient observed for Affymetrix GeneChip biological replicates (see for example [26]).

**Figure 3:**
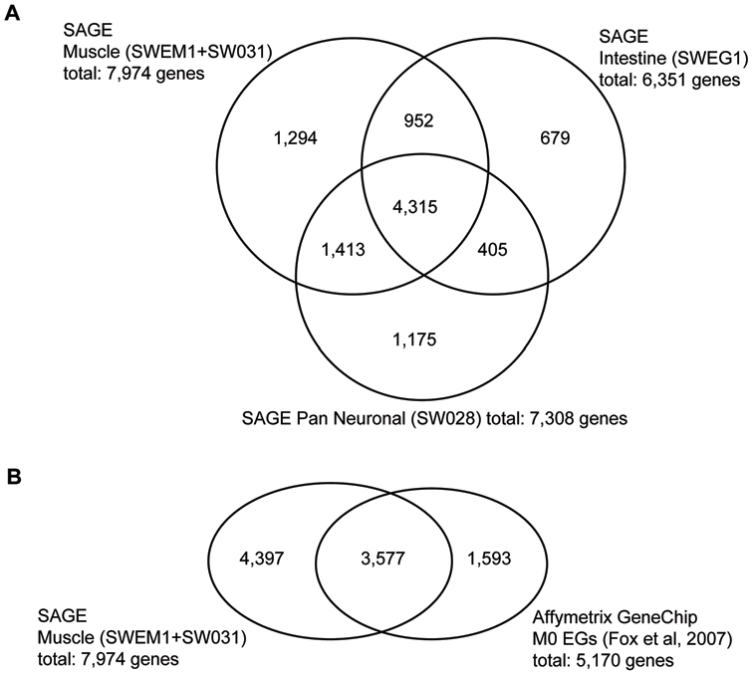
Comparison of the embryonic muscle SAGE profile with other tissue-specific SAGE libraries and Affymetrix GeneChip data. The overlap between genes found in tissue specific SAGE libraries for *C. elegans* muscle (SWEM1 and SW031), intestinal (SWEG1) and pan neuronal (SW028) cells are shown in (A). Only 11%–16% of the genes identified are unique for each cell type. (B) Comparison of both muscle SAGE libraries to previously published Affymetrix GeneChip data [26] on sorted embryonic muscle cells. Less than half (45%) of the 7,974 genes detected by SAGE were also detected by GeneChip technology.

Not all of the 7,974 genes we identified in the embryonic muscle libraries are necessarily specific to muscle, or even enriched in muscle. To gain some insight into these facets of expression we compared the results from the embryonic muscle libraries to those obtained from libraries using RNA isolated from purified embryonic intestinal cells (SWEG1) or pan neuronal cells (SW028) (Figure 3A, [35]; data available at http://elegans.bcgsc.bc.ca). We found that more than half of the muscle-expressed genes (4,315) are also present in both the intestinal and pan neuronal libraries, while about 15% of the genes (1,294) we detected are candidates for unique expression within muscle (Dataset S1). The remaining 2,365 genes are expressed in either neurons or the intestine in addition to muscle. Similar percentages of overlapping gene expression were observed for the other two tissues as well.

To identify genes with enriched expression in muscle we compared the relative abundance of sequence tags for a particular gene in the muscle libraries to the number of tags for the same gene in the whole-embryo SAGE library SWN22. A total of 192,661 sequence tags were obtained for the SWN22 library. Within this data set, there are 133,862 tags that pass the 0.99 sequence quality threshold representing 12,165 unique tag species and 9,064 genes (data available at http://elegans.bcgsc.bc.ca). Sequence tags for 6,984 of the muscle-expressed genes are also present in the whole embryo library. We used fold-change and a minimal tag count filter to determine enrichment. Depending on the fold-change cut-off that was used we observed between 1,459 (1.7 fold-change) and 228 (5.0 fold-change) genes enriched for expression in muscle (Dataset S2).

### A refined muscle transcriptome

Several different approaches have been taken to identify genes that are expressed in muscle cells. These include a genetic approach to isolate mutants with disorganized or non-functioning muscle, determining the cell expression patterns of proteins using antibodies or promoter GFP fusions, and identifying the transcripts present in isolated muscle cells by SAGE, GeneChip or rtPCR or cDNA analysis. Here we determine that the majority of the muscle genes identified by these other methods is present in our SAGE transcriptome.

Previous genetic analyses have identified about 70 genes that, when mutated, affect muscle structure or function in some way. Sixty-four of these known muscle-affecting genes are included in the 7,974 genes that define our muscle transcriptome (Dataset S3). Some muscle affecting genes that were not found in the muscle transcriptome encode proteins that are expressed in the hypodermis (*vab-19* and *mua-1*) or in neurons (*ace-2*) and not muscle [36]–[38], while others like *unc-22* are expressed in muscle cells but just not during embryogenesis [39]. A large-scale project involving the mapping of cell expression patterns of promoter GFP fusions has identified 587 genes that are expressed in muscle; data available at http://www.wormatlas.org/). We have determined that 452 of these genes (∼77%) are present in at least one of the two SAGE embryonic muscle libraries (Dataset S4). Interestingly, GFP expression in body wall muscle cells was observed for 39 genes that were represented by only a single SAGE tag and 167 genes that were represented by fewer than 5 SAGE tags in either SAGE library.

We also compared the muscle transcriptome generated by SAGE to one generated using an alternative gene expression analysis platform, the Affymetrix GeneChip [26]. The GeneChip study is comparable to our study as the analysis was also done using RNA isolated from FAC sorted embryonic muscle cells. The Fox et al (2007) study [26] identified 5,170 different transcripts in freshly sorted embryonic muscle cells after direct isolation at ‘0’ hours. A comprehensive list of 9,567 genes can be obtained by combining the SAGE and Affymetrix data; however, only 3,577 (37%) of these genes overlap the two data sets (Figure 3B; Dataset S5). As might be expected more highly expressed genes are more likely to be detected by both platforms. The mean sequence tag count for the genes detected by both SAGE and Affymetrix GeneChips is 19 tags per 100,000, but only 6 tags per 100,000 for the genes whose expression is detected by SAGE only. Similarly, the median sequence tag counts are 6 tags per 100,000 and 3 tags per 100,000, respectively. The moderate correlation (0.77) of SAGE sequence tag counts per gene and Affymetrix signal intensities for the 3,577 genes determined to be ‘present’ in both the SAGE and Affymetrix GeneChip data is consistent with previously published comparisons between SAGE and Affymetrix GeneChips [40].

Partially because of the large dynamic range of individual gene expression, (i.e. three orders of magnitude in our study) low abundance messages are a problem for all expression platforms to detect. They simply get swamped out by the more abundantly transcribed messages and are often lost during the stochastic process of sampling the RNA populations. For our SAGE studies we define a transcript as ‘present’ if it is represented by at least one sequence tag, even though there is some controversy as to whether singletons (i.e. single sequence tags representing a gene) are a reliable measure of gene activity [40]. The majority of the 4,397 genes that were identified in our SAGE study, but that were not identified in the Afffymetrix GeneChip study are low abundance transcripts with an average sequence tag count of 6 per 100,000.

Before initiating a functional study of the muscle transcriptome we felt it prudent to refine the transcriptome and only study genes with a high probability of actually being expressed in muscle cells. We have generated a refined embryonic-muscle transcriptome consisting of the 3,577 genes that are present in at least one of the two SAGE libraries and also in the GeneChip library generated by Fox et al, 2007 [26] (Figure 3B, Dataset S5).

### RNAi analysis of the muscle transcriptome identifies genes involved with sarcomere assembly and maintenance

Our refined embryonic-muscle transcriptome identified 3,577 genes that are expressed in muscle cells. A small number of these genes have been studied in great detail and much is known about their role in a functioning muscle cell (reviewed in [3]). However, little or nothing is known about the function and/or relevance to muscle development of the majority of the genes identified by transcriptional profiling. Gene inactivation or knockdown by RNAi has been used successfully to screen much of the *C. elegans* genome and possible functions have been suggested for some genes [41]–[43]. Rather than simply repeat previously performed RNAi screens, we have designed two muscle specific RNAi screens, one to identify genes essential for embryonic muscle formation, and another to identify novel genes involved in myofilament assembly. Both screens utilized the RNAi feeding library constructed by the Ahringer lab [42]. For various reasons we were not able to obtain double stranded RNA for all 3,577 genes in our muscle transcriptome, screening 85 and 92% of the genes respectively.

**Figure 4:**
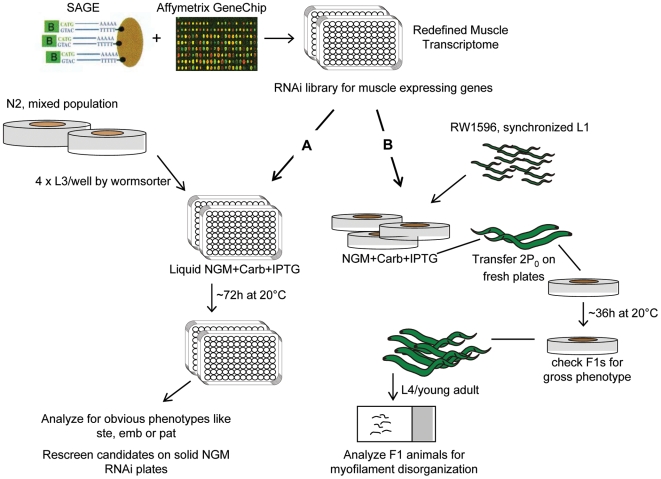
Schematic diagram for the two RNAi by feeding screens. The muscle transcriptome includes 3,297 genes that overlap with the Ahringer RNAi feeding library, the SAGE dataset and the Affymetrix GeneChip dataset. (A) The protocol used for a high-throughput liquid RNAi feeding screen to identify genes essential for embryonic muscle formation and (B) the protocol used for an RNAi feeding screen on solid media for defects in myosin localization using RW1596, a MYO-3::GFP expressing strain. See Materials and Methods for details.

**Figure 5:**
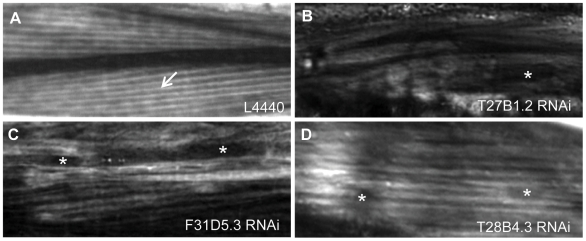
Body wall muscle structure in wild-type and RNAi-treated animals. (A) Myofilaments visualized by polarized light microscopy in wild-type animals treated with the empty RNAi feeding vector L4440 showing nicely organized filament structure (arrow). In animals treated with dsRNA for T27B1.2 (B), F31D5.3 (C) or T28B4.3 (D), filaments are disorganized and gaps are present in the myofilament (asterisk).

The purpose of our first RNAi screen was to identify genes essential for embryonic muscle formation. Previous work by Williams and Waterston (1994) [7] has shown that animals lacking functional muscle exhibit a similar embryonic arrest phenotype designated the Pat phenotype. To date, 19 genes that affect muscle have been shown to produce this phenotype when mutated [7], [9]–[12], [17], [44]–[51]. Here we utilized the approach outlined in Figure 4 pathway A to screen for Pat mutants after RNAi treatment of wild type animals. Our initial screen of 3,031 genes was done in liquid culture and identified 371 RNAi treatments resulting in embryonic lethality, sterility, or movement abnormalities. After eliminating well characterized and published genes, the remaining 170 positive genes were tested again using RNAi feeding on solid media. Further analyses revealed that four genes T27B1.2, F31D5.3, T28B4.3, and F25B3.6 consistently produced Pat animals when inactivated. We were also able to identify effects on post-embryonic muscle development by feeding double stranded RNA to wild type animals after hatching. Adult hermaphrodites were observed using polarized light microscopy and significantly disorganized body wall muscle was seen after RNAi treatment for the genes T27B1.2, F31D5.3, and T28B4.3 (Figure 5), but not F25B3.6 (data not shown).

**Figure 6:**
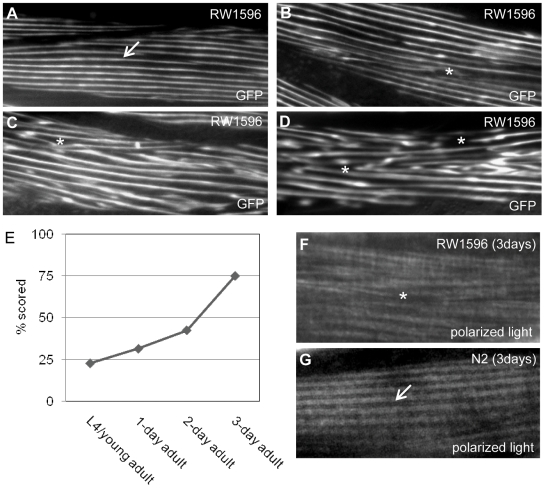
Sarcopenia in aging nematodes. Synchronized animals were observed for irregularities in filament organization with age using the GFP-tagged MYO-3 marker strain RW1596. Fluorescence images are shown for L4/young adult (A), 1-day adult (B), 2-day adult (C), and 3-day adult (D) animals. As the filament structure is nicely organized in L4/young adult animals (arrow), both the severity of myofilament disorganization (asterisk) as well as the percentage of animals affected increase during aging (E). To preclude any artifacts caused by GFP itself, we visualized disorganized filaments in 3-day adult RW1596 animals using polarized light (F, asterisk). No defects are visible in 3-day adult N2 animals (G, arrow), indicating an early onset of sarcopenia in RW1596 muscle filaments.

The second RNAi screen utilized the approach outlined in Figure 4 pathway B, and involved screening for defects in myosin localization, using the MYO-3::GFP expressing strain RW1596 [34]. The GFP-tagged MYO-3 protein in this strain is transcribed from a modified gene contained on a transgenic array and is the only *myo*-*3* gene product produced. Loss of the array results in animals that arrest at the two-fold stage of embryogenesis (the Pat phenotype). It has been shown previously that RW1596 animals experience an acute loss of myofilament stability with age [52]. To avoid false positive results in the screen, we first analyzed synchronized RW1596 worms from young adults until 3-days of adulthood for abnormalities in MYO-3::GFP expression. We found that the percentage of animals displaying myosin defects increased from 22.8% in the L4/young adult stage to 31.9% in 1-day old adults, 42.5% in 2-days old adults, and 75% in 3-days old adult animals. Figure 6 shows the observed myofilament abnormalities in aging RW1596 animals as visualized by GFP fluorescence microscopy (Figure 6A–6D) as well as polarized light microscopy (Figure 6F). This age related myofilament instability does not become apparent in N2 wild-type animals until much later (Figure 6G; our unpublished data). Thus, it would appear that the presence of the GFP-tagged MYO-3 protein renders the myofilaments more susceptible to perturbation than normal, providing a sensitized background for the detection of sarcomere affecting genes. As a result of this analysis, we screened only L4/young adult animals in the following RNAi experiment.

**Figure 7:**
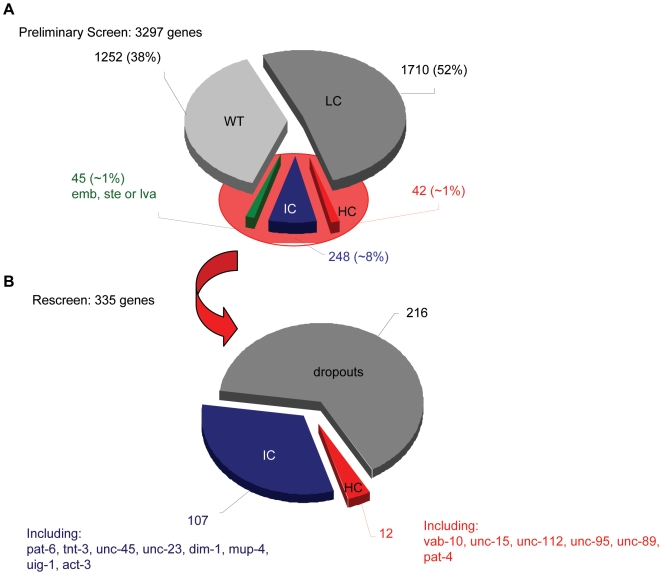
Diagram summarizing the results of the RNAi screen for defects in myosin localization. (A) The results of the preliminary screen were grouped into four classes according to the proportion of animals showing irregularities in myosin localization after RNAi treatment. *High class* (HC) for [75–100], *intermediate class* (IC) for [50–75], *low class* (LC) for [25–50], and *wild-type class* (WT) for [0–25] percent affected animals per scoring. Only genes resulting in IC (248 genes) and HC (42 genes) were considered as “muscle affecting” to be further analyzed in the re-screen. Feeding of 45 different RNAi constructs resulted in embryonic or adult lethality (green); no animals could be scored in the F_1_ generation and these genes were included in a modified re-screen (see Materials and Methods for details). (B) Summarized results of re-screening 335 genes identified as muscle affecting in the preliminary screen. Twelve genes remained in the HC category, 6 of which have been previously reported to affect *C. elegans* muscle structure, and 107 genes were scored in the intermediate class, including 7 known muscle-affecting genes. The remaining 216 genes did not reach the increased stringency and dropped under the 50% cutoff.

In a preliminary screen, we examined a total of 3,297 genes for RNAi-induced myofilament defects (Figure 7A). Briefly, adult hermaphrodites that had been grown on plates containing RNAi feeding bacteria were transferred to fresh plates containing the same bacteria for 24 hrs and then removed. Their progeny were scored as L4 larvae or young adults. After a brief inspection for overt phenotypic anomalies, animals were scored for myofilament defects and then assigned to one of four classes. In order for RNAi-treated hermaphrodites to be placed in the High class (HC), 75 to 100% of the animals screened had to exhibit some sort of MYO-3 abnormality. The proportions of affected animals for the Intermediate (IC), Low (LC) and Wild type (WT) classes were 50 to 75%, 25 to 50% and 0 to 25%, respectively. The majority of the genes that were tested caused either a Low (1,710) or a Wild type (1,252) class phenotype when inactivated by RNAi treatment. We only considered the RNAi treatments resulting in High or Intermediate phenotypes to be muscle-affecting. To confirm this data, we repeated the RNAi treatments for the 290 genes that fell in these two categories. In addition, a slightly modified screen (see Materials and Methods) was used to test the 45 essential genes identified in the primary screen that resulted in few, if any, adult progeny.

In the re-screen, both the number of analyzed animals and the scoring stringency were increased to reduce false-positive results. We required an animal to have myofilament defects in multiple muscle cells to be deemed affected. After this second round of screening, we identified a total of 118 genes affecting MYO-3::GFP localization and/or stability in the RW1596 strain (Figure 7B). The remaining genes failed to meet the imposed stringency of the second screen. The clones from the RNAi feeding library used for each of the 118 positive treatments were verified by PCR using gene specific primers, or in some cases, sequencing. Fifty-eight of the 118 genes that affect myosin localization and/or stability did not display any obvious anatomical or behavioral phenotypes when knocked down by RNAi and, therefore, would not have been identified in a conventional screen.

**Figure 8:**
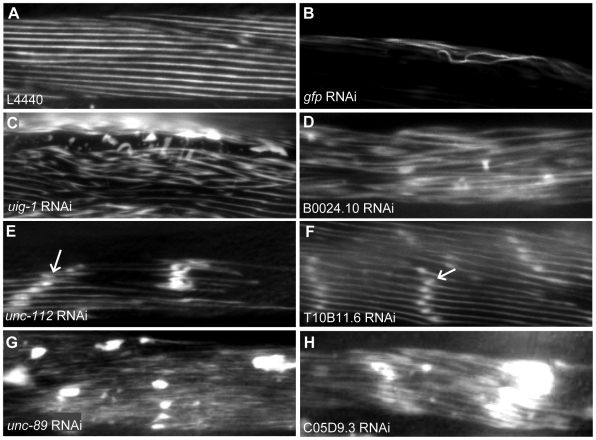
RNAi induced myofilament abnormalities visualized by MYO-3::GFP. Representative fluorescent images are shown for (A) the empty vector plasmid L4440, resulting in proper wild-type filament organization and (B) GFP RNAi resulting in strong reduction of GFP expression. The MYO-3::GFP abnormalities observed in the RNAi screen were grouped into three categories. (C) and (D) showing general disorganization of myofilaments with minor aggregations, (E) and (F) exhibit well ordered and small aggregations (arrow) along otherwise well organized filaments, and (G) and (H) large GFP clumps or deposits.

The MYO-3::GFP abnormalities observed have been arranged into three categories that are described below and in Dataset S6. In some cases gene inactivation by RNAi resulted in more than one of these myofilament abnormalities, albeit in different animals. This could be due to variability in gene knockdown from animal to animal. Figure 8A and 8B show the phenotypes observed for the control experiments that were included in this screen. The animal in Figure 8A was fed bacteria containing an empty L4440 feeding vector and represents the unaffected state. The MYO-3::GFP protein is well ordered in the myofilament lattice and there are no unlocalized clumps of GFP. The animal in Figure 8B was fed dsRNA for GFP and has a marked reduction in the amount of MYO-3::GFP protein produced. The most common phenotype observed after RNAi knockdown is a general disorganization of the myofilaments, including minor GFP aggregations, that somewhat resembles the age related sarcopenia observed in older RW1596 hermaphrodites. Examples of this phenotype are shown in Figure 8C and 8D. Eighty-two of the genes we tested have been assigned exclusively to this category (i.e. this was the only phenotype observed after RNAi knockdown), although most animals assigned to the two other categories also exhibited this phenotype in some muscle cells. The two other distinct phenotypes that we observed are less common. A total of 22 genes have been assigned to the second category. In muscle cells exhibiting this phenotype, the MYO-3::GFP containing filaments are well ordered but small aggregations of GFP appear along the filaments (shown in Figure 8E and 8F). In many cases large or small gaps in the lattice are also present indicating that some myofilaments have detached from the muscle cell membrane. Only 13 genes have been assigned to the third category. The MYO-3::GFP abnormalities in muscle cells exhibiting this phenotype are characterized by large GFP deposits, and are accompanied by disorganization of the myofilaments (shown in Figure 8G and 8H). Again large or small gaps in the lattice may also be present. Only one gene did not fall exclusively into any one category. Animals fed dsRNA for the known muscle gene *unc*-*112* displayed myofilament abnormalities consistent with both categories 2 and 3. The other 13 known muscle genes identified in this screen fall into all three phenotypic categories; *unc-45*, *uig-1*, *unc-23*, and *mup-4* were assigned to category 1, *dim-1*, *unc-95*, and *unc-15* were assigned to category 2 and *pat-4*, *pat-6*, *vab-10*, *unc-89*, *act-3*, and *tnt-3* were assigned to category 3 (see Dataset S6). All available information about our screen, including diagnostic images, may be accessed at http://www.zoology.ubc.ca/~alorch/rnai A subset of 22 genes that scored positive in the GFP reporter myofilament screen was also analyzed by RNAi in N2 wild-type animals using polarized light. The observed phenotypes in body wall muscle range from minor structural irregularities making it very difficult to visualize filaments under polarized light to severe disorganization (Figure S1). Six out of 22 genes did not result in a phenotype distinguishable from wild type filament structure using this approach and would therefore have been missed without the sensitized background.

Together our two RNAi screens have identified 108 genes not previously known to be involved in *C. elegans* muscle development and/or function (Dataset S6). Only 15 of the 122 genes we identified in our screens were previously characterized and shown to be required for proper muscle function. Working from GO annotation terms we have determined that several of these new muscle-affecting genes fall into the categories of cell structure/cellular processes, metabolism, gene expression, signal transduction, protein processing and protein-protein interactions. Several of these newly identified muscle-affecting genes have no assigned GO category and are, therefore, of completely unknown function. Based on Ensembl annotations (http://ensembl.org), about 58% of these new muscle-affecting genes encode proteins with sequence similarity to human proteins (Dataset S6). In some cases protein function can be inferred from the information known about the human ortholog. However, that still leaves many genes without a described function.

## Discussion

In this study we have combined two large scale technologies, transcriptional profiling using SAGE and gene inactivation using RNAi, to identify novel genes involved in myofilament assembly and/or stability. Our SAGE data identified 7,974 protein-coding genes that are expressed in embryonic muscle cells, whereas a very similar study using Affymetrix GeneChip technology identified 5,170 genes expressed in embryonic muscle [26]. In an attempt to avoid sampling bias and low-level contamination by non-muscle transcripts we chose to focus on genes identified by both expression platforms; these would, presumably, have a higher probability of being expressed in muscle cells. Our more limited embryonic muscle transcriptome consists of ∼3,500 genes, and we used gene inactivation or knockdown by RNAi to determine the function of some of these genes.

We generated two replicate long SAGE libraries for sorted embryonic muscle cells, identifying 7,974 protein-coding genes, including 64 previously described muscle affecting genes. More than half of the genes identified by SAGE were not detected using GeneChip technology, although the majority of these are represented among the 22,150 transcripts represented on the Affymetrix *C. elegans* genome array [26]. Conversely, about one third of the genes detected by the GeneChip microarray are missing form the SAGE libraries. Presumably a large portion of this discrepancy is due to sampling and distinguishing signal from noise. Consistent with previously published comparisons most of the genes detected by SAGE but not detected by GeneChip technology (∼68%) have fewer than 5 sequence tags/library [40]. The total number of genes with only one sequence tag (singletons) in either SW031, SWEM1 or both libraries accounts for 31.4% of the total number of genes identified by SAGE analysis. In contrast, less than 19% of the total number of genes identified by both SAGE and GeneChips are singletons, 675 of the 2504 singletons (27%) identified by SAGE are present in the Affymetrix GeneChip study. In addition, we confirmed a number of singletons and genes with low tag counts (<5) to be present in muscle cells by GFP reporter studies. These data demonstrate that a not insignificant number of genes are transcribed at very low levels in muscle and one cannot simply ignore these low level transcripts if one wishes to determine the full transcriptome of any specific tissue or cell type. In future deep sequencing SAGE libraries may be the best way to surmount the difficulty of gene expression across four orders of magnitude. Low-level contamination will still be a problem but at least detection will not be an issue.

As expected, many of the genes identified in embryonic muscle also function in several types of cells. Comparing the data obtained from the muscle, intestinal and pan neuronal libraries revealed that between 11–16% of the genes identified in any one tissue are specific to that tissue, although these estimates are possibly high. Some of the genes in the muscle embryonic library are enriched in muscle when compared to the other tissue specific libraries or the whole embryo library. Generating SAGE library expression profiles is useful for identifying candidate muscle genes for further functional studies. We have identified 228 genes enriched 5-fold or more in embryonic muscle cells compared to the whole embryo, including known genes like *mup-2* (troponin [49], 21.8-fold), *unc-95* (LIM-domain containing protein [14], 7.7-fold), and *mlc-2* (myosin light chain [53], 14.3-fold) as well as many unknown or uncharacterized genes (Dataset S2). Lowering the fold-change cut-off to 1.7 reveals 1,459 muscle-enriched genes.

Our RNAi screens identified 108 new muscle-affecting genes. Only four of these were identified in the first screen that specifically looked for genes required for embryonic muscle function. Many embryonic lethal mutants have been found in various RNAi screens [43],[54], but until now there has not been a screen focused solely on identifying animals exhibiting the Pat phenotype. In fact, two of the four genes found in our study (F31D5.3 and T28B4.3) have not been reported to cause embryonic lethality in any previous RNAi screens. Several genetic studies have identified at least 19 loci that encode proteins essential for muscle formation during early development (reviewed in [3]). Most of these genetic loci, with the exception of *pat*-*9* and *pat*-*11*, have been identified at the sequence level and thus are correlated with the physical map. Seventeen of the 19 known Pat genes were included in our muscle transcriptome, and our screen identified only eight of these. Given these results it would seem that RNAi knockdown by feeding is not a very efficient method for identifying Pat mutants. If this is the case, there are probably more “*pat*” genes to be discovered. Injection of double stranded RNA instead of feeding has been used quite successfully by others [54],[55] and our preliminary RNAi knockdown treatments using this method suggests that it is a much more effective, but also more time consuming and expensive way to identify Pat mutants.

The majority of the new muscle-affecting genes that were discovered were identified by specifically screening our refined embryonic muscle transcriptome for disorganized myosin filaments using a MYO-3::GFP marker strain, RW1596 [34]. The only functional myosin heavy chain A (MYO-3) protein produced in these animals is fused to GFP, a bulky, tubular protein composed of 238 amino acids [33]. We have discovered that the myofilaments in the body wall muscle of RW1596 hermaphrodites are more sensitive to perturbation and the effects of aging than those in the body wall muscle of wild type hermaphrodites. Presumably, the barrel-shaped GFP reporter fused to MYO-3 prevents the tight packing of these molecules in the central bipolar H zone of thick filaments. This would negatively affect the levels of myosin heavy chain B (UNC-54) and paramyosin (UNC-15), as well as prevent the proper assembly of these molecules into a functional thick filament [56]. Thus, it is very likely that GFP-tagging of the MYO-3 protein results in looser packing of the myosin rods, thereby making the strain more sensitive to the absence of auxiliary proteins resulting from RNAi treatment.

It should be noted that several genes previously shown to be required for proper muscle assembly and maintenance were not identified in this muscle RNAi screen. For example, treatment of animals with *pat-3*/ß-integrin dsRNA, despite having displayed both sterility and uncoordinated movement, did not exhibit a highly penetrant disorganized myofilament phenotype, and therefore it was assigned to the LC class. There are several possible explanations for not detecting previously described muscle affecting genes, including the inherent variability in RNAi screening, loss of the genomic insert in the bacterial clone, or the means by which we screened animals for myofilament defects.

Validation of our approach was provided when we identified several known muscle-affecting genes in the RNAi screens (Dataset S6). For example, the UIG-1 protein was identified as a novel dense body protein in a yeast two-hybrid screen for UNC-112 interacting proteins [57]. Although animals homozygous for the *uig*-*1*(*ok884*) null mutation appear healthy and fertile, disorganization of the body wall muscle filaments was observed in a polarized light assay [57]. In our RNAi screen, as well as in previous RNAi screens [43],[54],[58],[59], knockdown of the *uig-1* gene by RNAi treatment did not result in any obvious morphological or growth phenotypes. However, in the myofilament screen we were able to detect MYO-3::GFP disorganization in 55% of scored animals (n = 63), resulting in an intermediate class phenotype, and this phenotype correlates nicely with the earlier results [57]. The *uig-1* gene is an example of a gene that affects muscle organization but does not display a Pat or Unc phenotype. Such genes would be difficult to identify in a high throughput screen without a reporter gene for sarcomere integrity. In total, we identified 58 genes without an obvious growth or anatomical phenotype when inactivated by RNAi treatment, but which displayed an intermediate or high class phenotype when investigated for MYO-3::GFP localization. While some of these genes may encode proteins involved in muscle sarcomere assembly, we suspect most are involved in accessory functions involved with sarcomere maintenance and stability. Screening specifically for myofilament abnormalities, therefore, has proven to be an effective way to identify new genes affecting body wall muscle structure and function.

The myofilament abnormalities we have observed in our screen range from small aggregates to large deposits of GFP, which may be within the filaments or adjacent to them. We also see gradients of disorganization ranging from mild discontinuities in the filaments to large interruptions often accompanied by the accumulation of the MYO-3::GFP reporter protein. On occasion, we also observed a marked decrease of MYO-3::GFP signal in the myofilaments. These defects may be the result of several factors, including abnormal or aberrant localization of MYO-3::GFP, early onset sarcopenia, loss of myofilament stability and/or defective myofilament assembly. With one exception (*unc*-*112*), we assigned the 118 genes identified in our screen to different categories based on the myofilament phenotype observed after RNAi knockdown (Dataset S6). The first category represents the least severe phenotype observed and includes the majority of the genes identified, although only 4 of the 14 known muscle-affecting genes were assigned to this category. This phenotype is consistent with an early onset of the age related myofilament disorganization that is seen in older RW1596 and wild type animals. However, our current data do not allow to us to determine whether the disorganization observed is due to defective myofilament assembly, loss of myofilament stability or perhaps both. Two of the 4 known genes in this category encode molecular chaperones and another encodes a signaling molecule. The third category represents the most severe phenotype observed and has the fewest assigned total genes (14) but half of the known muscle affecting genes (7/14). The majority of the known genes assigned to category three encode structural components of dense bodies and/or M-lines or the myofilaments, and most have been shown to be essential for the assembly of the sarcomere during embryogenesis.

Annotation of our new candidate muscle-affecting genes using ENSEMBL identified many different functions. Obviously, genes encoding structural proteins or those with protein binding domains are good candidates to be involved in sarcomere assembly and/or stability, but predicted metabolic or signaling molecules can have a structural function as well, as observed for *pat-4*, *dim-1* and *uig-1* [11],[16],[57]. We also identified seven genes involved in protein turnover that appear to cause defects in sarcomere structure. It has been shown previously that the degradation of UNC-45, a molecular chaperone for myosin, by the E3/E4 complex formed by CHN-1 and UFD-2 is indispensable for proper myosin folding and assembly into thick filaments [60],[61]. These newly identified genes may be involved in comparable pathways.

Using integrated strategies like SAGE and RNAi provides an initial step towards a comprehensive analysis of all the genes required to form and maintain a muscle sarcomere in *C. elegans*. A recent RNAi study from the laboratory of Norbert Perrimon using Drosophila primary culture cells is, in principal, similar to our study [62]. They identified 49 genes involved in late muscle differentiation after examining 1,140 randomly chosen genes, and 22 of these genes were not previously known to be involved in muscle function. Thirty-five of the 49 muscle-affecting genes identified in the Drosophila RNAi study have a *C. elegans* ortholog. However, only 16 of these met the SAGE and microarray criteria of our study, and only 2, *unc*-*15* and *unc*-*112*, were identified as affecting myofilament organization. The remaining genes were assigned to either the low class (9) or the wild type class (5). The data obtained in these two studies reveals a significant number of new players involved in muscle development. With the identification of so many new protein involved in sarcomere formation and maintenance perhaps we are nearing the time where some of the more outstanding problems concerning sarcomere assembly can be solved.

## Material and Methods

### *C. elegans* strains

All strains were maintained using standard culture methods [63]. The following strains were used: **N2:** wild-type Bristol isolate, **RW1596:** *myo-3(st386)*V; *stEx30* [34].

### FACS analysis

GFP expressing muscle cells were isolated from freshly dissociated RW1596 embryos as described previously [64] with minor modifications. Embryos were treated with Chitinase (0.5 units) to dissolve the eggshell, concentrated by high-speed centrifugation and then repeatedly passed through a 21-gauge needle to dissociate the cells. The resulting cell suspension was filtered through a 5 µm Millex-SV syringe filter, and pelleted by gentle centrifugation. Isolated cells were resuspended in ice-cold egg buffer and maintained on ice in preparation for the following FACS procedure.

GFP expressing embryonic muscle cells were isolated using a FACSVantage SE Diva cell sorter, equipped with a 488 nm Argon laser and a 530±15 nm emission filter for GFP, and a 585±22 nm emission filter for propidium iodide (used to discriminate dead cells). Non-GFP embryonic cells from wild type N2 worms were used as controls to set instrument settings, and to establish GFP sorting gates. Initially cells were analyzed for light scatter properties such that a population of cells in a forward scatter (cell size) vs. side scatter (granularity) plot was selected that eliminated clumps and debris. This population was then analyzed in a GFP vs. PI plot to visualize GFP positive cells. The GFP sorting gates were defined by comparing the profile of wild-type embryonic cells with that of the MYO-3::GFP expressing cells. Sorted cells were collected in 0.5 ml of egg buffer supplemented with 5% FBS and immediately frozen in liquid nitrogen in preparation for RNA isolation. An aliquot of cells from each sort was cultured overnight on peanut lectin coated cover slips with L-15 and 10% FBS as a culture medium. This overnight culture was examined using fluorescent-light microscopy to gauge sort purity. Library construction involved pooling RNA recovered from sorts that were deemed to have a purity of ≥90%.

### Production and analysis of the SAGE libraries

SAGE data for SWEM1, SW031, SWEG1, SW028, SWN22 were prepared by standard methods and analyzed as described elsewhere [65],[66]. All information is available at http://elegans.bcgsc.ca/home/sage.html; Wormbase version WS180 (September 2007) was used for gene identification.

### RNAi screen for essential muscle genes in liquid culture

The N2 strain was used for these experiments following the protocol described previously [67]. RNAi clones were grown in 96-well plates overnight at 37°C in 150 µl of L-broth containing 50 µg/ml carbenicillin and 8% glycerol. The following morning, 10 µl of each freshly grown bacterial stock was transferred to 96-well plates of Liquid NGM media (50 µl per well) containing 4 mM IPTG and 50 µg/ml carbenicillin. Liquid NGM plates were incubated for 16 hours at 20°C and then 37°C for 2 hours. Four synchronized L3 worms were then transferred into each well using a Copas Biosorter-250 (Union Biometrica) and incubated at 20°C for 72 hours. Each well was scored for animals that exhibited embryonic lethality, paralysis, or sterility. All RNAi treatments that resulted in animals with the aforementioned phenotypes were re-screened using solid NGM media. RNAi bacterial cultures were grown overnight in 96-well plates containing 200 µl of L-broth with 50 µg/ml carbenicillin. NGM plates containing 1 mM IPTG and 50 µg/ml carbenicillin and were then streaked with the freshly grown RNAi bacterial cultures and incubated at 20°C for 24 hours. Two L4 animals were then transferred to each plate, and incubated for 72 hours at 20°C. Two F_1_ progeny at the L4 stage were then transferred to each of three replicate plates for each RNAi treatment, and incubated at 20°C for 24 hours to lay eggs. All of the plates were examined for animals exhibiting an early embryonic arrest phenotype, a mixed stage embryonic arrest phenotype, or a two-fold stage arrest phenotype.

### RNAi screen for myofilament defects

The strains RW1596 and N2 wild-type was used for these experiments following the protocol described previously [41]. RNAi colonies were grown overnight in L-broth with 50 µg ml^−1^ ampicillin and then streaked onto NGM plates containing 1 mM IPTG and 50 µg ml^−1^ carbenicillin. The plates were incubated overnight at room temperature to induce dsRNA expression. Approximately 30 synchronized L1s (P_0_) animals were spotted onto each RNAi plate. These plates were incubated at 20°C until the worms reached the young adult stage (∼60–68 hours), then 2 P_0_ were transferred onto fresh plates, in duplicate for each RNAi clone. To synchronize the F1 generation, the adult animals were removed after an 18-hour egg-laying period. Embryos were allowed to hatch and develop to the L4/young adult stage (∼36 hours). Prior to the myofilament screen, animals were scored for a variety of gross anatomical and growth defects such as sterility, embryonic or larval lethality, uncoordinated movement, and slow growth. Then, a random subset of worms (10–40) from each duplicate plate were picked into 15 µl M9 buffer containing 10% sodium azide and their body wall muscle was screened for MYO-3::GFP defects using a compound fluorescence microscope (Zeiss Axiophot). An animal was scored positive when the majority of muscle cells showed abnormal GFP fluorescence. Animals were examined blind to the identity of the dsRNA. The empty vector (L4440) was used as a negative control, and worms feeding on bacteria expressing dsRNA for *unc-97* or GFP were used as positive controls on each day of screening. Digital photographs were taken using a Qimaging QICAM digital camera running Qcapture version 1.68.4.

For clones resulting in an embryonic lethal or sterile phenotype in the first screen, the RNAi screening protocol described above was modified as followed. The synchronized L1 worms were spotted onto empty vector (L4440) RNAi control plates and then transferred to gene specific RNAi plates after reaching the adult stage. The F1 generation was then examined for myofilament defects as L4/young adults. All essays were carried out at 20°C.

### Database for myofilament screen

Images and observations for each gene screened were archived in an online database, created using MySQL 3.23.49 with a web interface written in PHP 4.3.4. Hosting was set up on a server maintained by the Department of Zoology, University of British Columbia (Canada).

## Supporting Information

Figure S1 RNAi induced myofilament abnormalities visualized by polarized light. (A) Myofilaments visualized by polarized light microscopy in wild-type animals treated with the empty RNAi feeding vector L4440 showing nicely organized filament structure (arrows). In animals treated with dsRNA for F52A8.1 (B) and H15N14.2 (C) filaments are disorganized and inconsistent (asterisk). Filaments in animals treated with dsRNA for T06A10.3 (D) are showing structural abnormalities at the cell-cell boundaries (arrowheads).
(10.06 MB TIF)

Dataset S1 Comparing muscle, intestinal, and pan neuronal SAGE libraries.
(5.74 MB XLS)

Dataset S2 SAGE tags comparing muscle versus embryo.
(5.93 MB XLS)

Dataset S3 SAGE tags for known muscle affecting genes.
(0.10 MB XLS)

Dataset S4 Comparison muscle SAGE and promoter::GFP expression in muscle.
(1.06 MB XLS)

Dataset S5 Comparing two methods, SAGE versus Affymetrix GeneChip.
(5.22 MB XLS)

Dataset S6 A comprehensive list of all genes identified in both RNAi screens.
(0.23 MB XLS)
