# Supplementary figures and images for: An Integrated Strategy to Study Muscle Development and Myofilament Structure in Caenorhabditis elegans
Source: PLoS Genet. 2009 Jun 26;5(6):e1000537. doi: 10.1371/journal.pgen.1000537 (PMC2694363; doi:10.1371/journal.pgen.1000537)

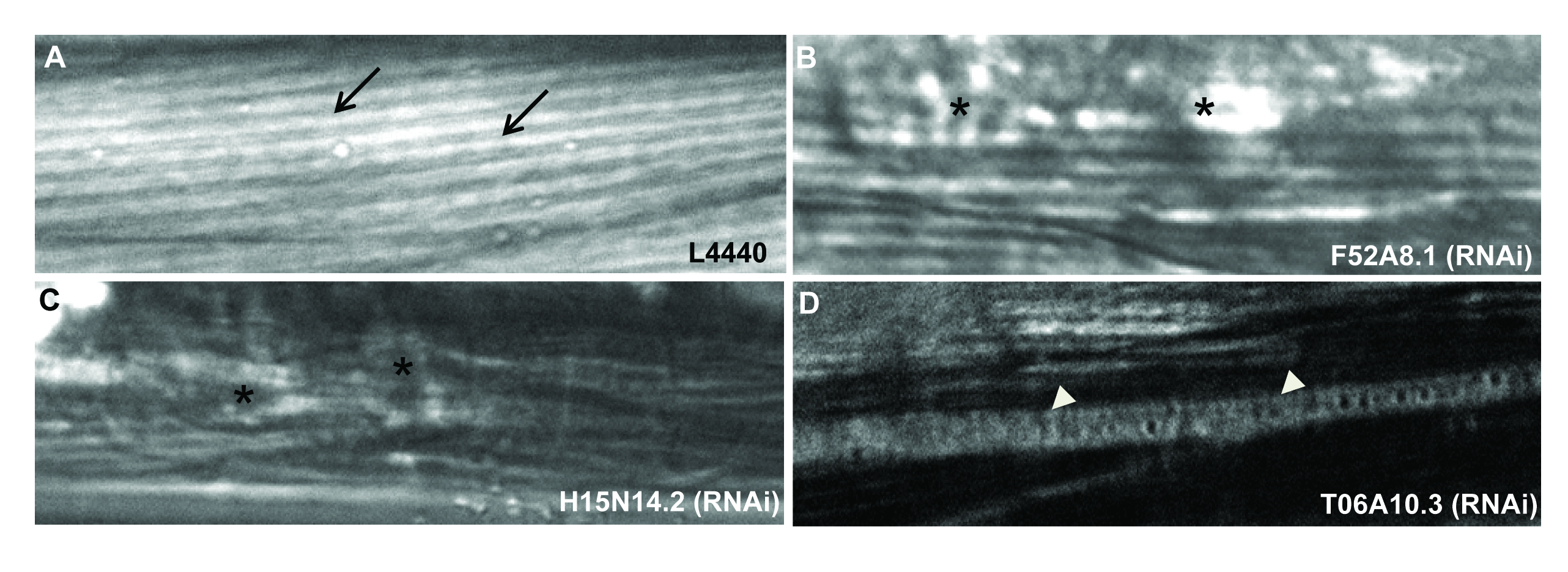

Supplement: Figure S1 — RNAi induced myofilament abnormalities visualized by polarized light. (A) Myofilaments visualized by polarized light microscopy in wild-type animals treated with the empty RNAi feeding vector L4440 showing nicely organized filament structure (arrows). In animals treated with dsRNA for F52A8.1 (B) and H15N14.2 (C) filaments are disorganized and inconsistent (asterisk). Filaments in animals treated with dsRNA for T06A10.3 (D) are showing structural abnormalities at the cell-cell boundaries (arrowheads). (10.06 MB TIF) [file pgen.1000537.s001.tif]
